# The Impact of Depressive Symptoms on Emotional Sensitivity in Early‐Stage Alzheimer's Disease: Implications for Social and Emotional Well‐Being

**DOI:** 10.1002/alz70857_106130

**Published:** 2025-12-25

**Authors:** Bailey L Ortiz, Angelina M Quagletti, Liberty Hebron, Bruce L. Miller, Joel H Kramer, Gil D. Rabinovici, Katherine P Rankin

**Affiliations:** ^1^ Memory and Aging Center, Weill Institute for Neurosciences, University of California, San Francisco, San Francisco, CA, USA; ^2^ Palo Alto University, Palo Alto, CA, USA; ^3^ Palo Alto University, 94304, CA, USA; ^4^ Global Brain Health Institute, University of California, San Francisco, San Francisco, CA, USA; ^5^ UCSF Alzheimer's Disease Research Center, San Francisco, CA, USA; ^6^ Memory and Aging Center, UCSF Weill Institute for Neurosciences, University of California, San Francisco, San Francisco, CA, USA; ^7^ Global Brain Health Institute, University of California San Francisco, San Francisco, CA, USA; ^8^ Memory and Aging Center, Weill Institute for Neurosciences, University of California, San Francisco (UCSF), San Francisco, CA, USA; ^9^ Molecular Biophysics and Integrated Bioimaging Division, Lawrence Berkeley National Laboratory, Berkeley, CA, USA; ^10^ Department of Radiology and Biomedical Imaging, University of California San Francisco, San Francisco, CA, USA; ^11^ Memory and Aging Center, Weill Institute for Neurosciences, University of California San Francisco, San Francisco, CA, USA; ^12^ Global Brain Health Institute (GBHI), University of California San Francisco, San Francisco, CA, USA

## Abstract

**Background:**

Emotion sensitivity, including emotion reading and real‐life empathy, may be altered in Alzheimer's disease (AD), with some individuals experiencing impairments while others show heightened sensitivity. Depression, common in early AD, may influence empathy and social cognition, yet the impact of depression on emotion sensitivity remains unclear. Clarifying these relationships could inform interventions to support social and emotional well‐being in early AD.

**Method:**

252 older adults with early‐stage AD (Clinical Dementia Rating 0.5–1; 52% female) self‐reported depressive symptoms using the Geriatric Depression Scale (GDS) across five subscales (Dysphoria, Hopelessness, Worry, Withdrawal, Cognitive Concern), and underwent emotion comprehension testing with the Dynamic Affect Recognition Test (DART) and The Awareness of Social Interference Test (TASIT) Emotion Evaluation Test (EET). Informants described participants’ empathic functioning using the Interpersonal Reactivity Index (IRI), measuring Perspective‐Taking, Fantasy, Empathic Concern, and Personal Distress. Univariate and multivariate regression models examined associations between depressive symptoms and emotion sensitivity, adjusting for age, sex, and education.

**Result:**

Regression modeling showed that negative mood (Dysphoria and Hopelessness) hindered emotional connection and cognitive empathy (Empathic Concern: β=‐0.65, *p* <0.05; β=‐0.84, *p* <0.05; Perspective‐Taking: β=‐0.87, *p* <0.001; β=‐1.39, *p* <0.05). Greater Withdrawal predicted lower empathic Perspective‐Taking (β=‐0.62, *p* <0.05), suggesting social disengagement impairs considering others' viewpoints. Worry and Cognitive Concern predicted greater Emotion Comprehension (β=0.40, *p* <0.05; β=0.26, *p* <0.05) but lower Empathic Concern (β=‐1.34, *p* <0.001; β=‐0.54, *p* <0.05), showing that patients with these mood states more accurately identify others emotional expressions while still struggling to engage empathically. Finally, individuals with greater Hopelessness felt more overwhelmed in social interactions (Personal Distress: β=1.23, *p* <0.05).

**Conclusion:**

Depression significantly impacts both emotion reading and real‐life empathy in AD, including empathic concern and perspective‐taking. Negative mood (dysphoria and hopelessness) hindered emotional connection despite having no effect on emotion reading. Increased mental rumination (worry and concerns about cognition) was linked to reduced empathic engagement, despite heightened emotion comprehension skills. These complex relationships underscore the importance of identifying the specific nature of depressive symptoms when evaluating individuals with early‐stage AD, to better implement targeted interventions that preserve social engagement and emotional well‐being.

